# A statistical simulation model to guide the choices of analytical methods in arrayed CRISPR screen experiments

**DOI:** 10.1371/journal.pone.0307445

**Published:** 2024-08-20

**Authors:** Chang Sik Kim, Jonathan Cairns, Valentina Quarantotti, Bogumil Kaczkowski, Yinhai Wang, Peter Konings, Xiang Zhang

**Affiliations:** 1 Data Sciences & Quantitative Biology, Discovery Sciences, BioPharmaceuticals R&D, AstraZeneca, Cambridge, England; 2 Functional Genomics, Discovery Sciences, BioPharmaceuticals R&D, AstraZeneca, Cambridge, England; Augusta University, TAIWAN

## Abstract

An arrayed CRISPR screen is a high-throughput functional genomic screening method, which typically uses 384 well plates and has different gene knockouts in different wells. Despite various computational workflows, there is currently no systematic way to find what is a good workflow for arrayed CRISPR screening data analysis. To guide this choice, we developed a statistical simulation model that mimics the data generating process of arrayed CRISPR screening experiments. Our model is flexible and can simulate effects on phenotypic readouts of various experimental factors, such as the effect size of gene editing, as well as biological and technical variations. With two examples, we showed that the simulation model can assist making principled choice of normalization and hit calling method for the arrayed CRISPR data analysis. This simulation model is implemented in an R package and can be downloaded from Github.

## Introduction

Application of Clustered Regularly Interspaced Short Palindromic Repeats (CRISPR) and the CRISPR-associated (Cas) proteins has enabled investigating genetic perturbations at genome-scale [1]. Arrayed CRISPR screens are primarily used for validation and follow-up investigations after whole genome pooled CRISPR screening [2–4]. Since the research question focus on ranking candidate genes based on their phenotypic effect, in arrayed CRISPR screens, gene-specific oligonucleotide sequences (i.e., guide RNAs) are introduced to different wells in the microplates (such as 384-well plates) to knockout different genes. To study the phenotypic effects of different gene knockouts, the arrayed CRISPR screening experiment is often analyzed by high-content imaging based on a single or multiple immunofluorescent biomarkers [5].

Despite the routine use of arrayed CRISPR screens in industry and academics labs, there is no best practice for the data analysis of the arrayed CRISPR screening experiments. In general, normalization and hit calling are two important analytic procedures in the arrayed CRISPR screening data analysis [6]. This is because the arrayed CRISPR screening experiments are often affected by technical artefacts such as batch effect and spatial bias in the readouts. In particular, spatial bias can be caused by factors such as uneven temperature, humidity, or liquid dispensing [7], leading to a dependency between readout values and the location of the well in the plate. Normalization methods such as LOESS [8] and B-scores [9] are two statistical methods that can be used to mitigate the spatial bias. For hit calling, t test and linear mixed effect models are two possible options. In particular, the t test compares the mean readout value of tested gene knockout wells to the mean readout value of the neutral control wells. The “neutral control” refers to gene knockout that is known not to change the phenotype. The linear mixed effect (LME) model [10] is useful to account for variation between batches when multiple plates are used in an arrayed CRISPR screening experiment. In addition to the methods mentioned above, there are other normalization and hit calling methods. Importantly, poor choices of normalization and hit calling methods can lead to false positive or false negative findings [11]. It is important for scientists to be aware what is a good or “good enough” choice. So far data analysis workflow benchmark efforts have been focused on the pooled CRISPR screening [12–18]. However, that benchmark cannot indicate which algorithm is good for the arrayed CRISPR screen, since the pooled CRISPR uses a different readout (sequencing read count).

The rapid progression in gene editing technologies has led to an urgent demand for advanced computational tools to decipher the intricate datasets these methods produce. While simulation tools for gene expression data from microarray or RNA-seq platforms exist [19, 20], the fundamental model assumptions inherent to each differ markedly. The simulation tool we introduce is specifically engineered for high-throughput arrayed CRISPR screen data, encapsulating distinctive model assumptions that capture the complexities of CRISPR-mediated perturbations and their consequent impact on endpoint measurements. Existing tools, such as CRISPulator [21] and CRISPhieRmix [16], cater to pooled CRISPR screen experiments. Yet, to the best of our knowledge, there is a gap in published research that tackles the simulation of arrayed CRISPR screen experiment data directly. This highlights the need for further research to better understand the data analysis workflow for arrayed CRISPR screen datasets. In this study we present a statistical simulation model that can help scientists to make a principled choice for what normalization and hit calling method to be used for the arrayed CRISPR screening data analysis.

In the following section, we are going to first present the details of this simulation model. After that we are going to show that our model can generate synthetic arrayed CRISPR screening data with features similar to the real-life experimental data. Then we are going to present two examples on how the simulation model helped us to make a principled choice of normalization method and a preferred computational workflow for the arrayed CRISPR screening data.

## Methods

### Simulation model

We design our simulation model to cater for a typical arrayed CRISPR screen, with the aim to identify genes that have a significant effect on the phenotype of interest. For this purpose, we assume that the screen uses 384-well plates and applies image analysis to measure specific immunofluorescent biomarker expression on the cellular surface. A well-level summary measure is then used for downstream analysis. By default, the image analysis software (e.g., PerkinElmer’s Columbus) calculates this summary measure for each well by calculating the mean fluorescent intensity across all the single cell measurements in that well.

$$y\left[i,j\right]=\frac{\sum_{k=1}^{{\text{N}}_{\text{cell}}\left[i,j\right]}y_{\text{per}\;\text{cell}}\left[i,j\right]\left[k\right]}{{\text{N}}_{\text{cell}}\left[i,j\right]}$$ (1)

*y*[*i*, *j*] represents the mean biomarker intensity value in the well located at row *i* and column *j*. N_cell_[*i*, *j*] is the number of cells in the well located at *i*th row and *j*th column. Since the cell count must be an integer and varies between different wells due to technical factors such as pipette, we assumed that N_cell_[*i*, *j*] follows a Poisson distribution:

$${\text{N}}_{\text{cell}}\left[i,j\right]∼\text{Poisson}\left(\mu_{{\text{N}}_{\text{cell}}}\right)$$ (2)

In a previous study [22], Poisson distribution was used to model the number of cells per well. $\mu_{{\text{N}}_{\text{cell}}}$ is the average number of cells per well and usually pre-defined by the lab scientists. As genes that directly affect cell viability (“essential genes”) are usually undesirable in drug discovery, we assume that these genes have been removed from the screen–that is, we assume that the genes had no effect on the cell counts. *y*_per cell_[*i*, *j*][*k*] represents the biomarker expression value of the *k*th cell in the well located of the row *i* and column *j*. We assume that the fluorescent intensity *y*_per cell_[*i*, *j*][*k*] follows a lognormal distribution:

$$y_{\text{per}\;\text{cell}}\left[i,j\right]\left[k\right]∼\text{Lognormal}\left(z_{\text{per}\;\text{cell}}\left[i,j\right]\left[k\right],\sigma_{\text{tech}}\right)$$ (3)

We chose the lognormal distribution to ensure that *y*_per cell_[*i*, *j*][*k*] is positive, and a previous study [23] showed that lognormal was an appropriate distribution for fluorescence intensity values. Here, *σ*_tech_ represents the measurement error, and it is on the log scale. This parameter corresponds to photon noise and read noise in fluorescence microscopy. *exp*(*z*_per cell_[*i*, *j*][*k*]) represents the true signal of the *k*th cell in the well located at *i*th row and *j*th column. Our model assumed that the true signal is a sum of the biological signal (*x*_per cell_[*i*, *j*][*k*]) and the non-biological systematic error (Spatial Bias[*i*, *j*]):

$$exp\left(z_{\text{per}\;\text{cell}}\left[i,j\right]\left[k\right]\right)=x_{\text{per}\;\text{cell}}\left[i,j\right]\left[k\right]+\text{Spatial}\;\text{Bias}\left[i,j\right]$$ (4)

Here *x*_per cell_[*i*, *j*][*k*] and the Spatial Bias[*i*, *j*] are on the original scale. Spatial bias is characterized as under- or overestimation of measurements in certain rows and columns in a plate. We assume that it is caused by environmental factors such as irregular changes in the temperature, incubation time, and humidity, or technical factors such as pipette and plate reader. Traditionally, the spatial bias is modeled as an additive effect to the biological signal [24]. We followed the same modeling strategy and assumed that the spatial bias is the same for all cells in the same well.

Within a single well, there are N_cell_[*i*, *j*] cells in the same condition. To account for the variation between different cells, we assume that the biological signal *x*_per cell_[*i*, *j*][*k*] follows a normal distribution:

$$x_{\text{per}\;\text{cell}}\left[i,j\right]\left[k\right]∼\text{Normal}\left(\mu_{\text{per}\;\text{cell}}\left[i,j\right],\sigma_{\text{cell}}\right)$$ (5)

*σ*_cell_ represents the between cell variation in the biomarker expression level and it can be caused by different editing efficiency in different cells. *μ*_per cell_[*i*, *j*] represents the single-cell biomarker expression level in a particular condition that is applied to well at *i*th row and *j*th column. As *μ*_per cell_[*i*, *j*] must be a positive value, we model it as

$$log\left(\mu_{\text{per}\;\text{cell}}\left[i,j\right]\right)=\alpha +\beta_{G}\left[g\right]\text{G}\left[i,j\right]+\left(\beta_{T}+\beta_{TG}\left[g\right]\text{G}\left[i,j\right]\right)\text{T}\left[i,j\right]$$ (6)

*α* is the biomarker expression level on the log scale when there is no gene knockout nor other treatment. *β*_*G*_[*g*] is the gene editing effect for gene *g*. *β*_*T*_ is the treatment effect from a given drug. *β*_*TG*_[*g*] is the interaction between editing gene *g* and treatment. Because this is a linear regression model on the log transformed outcome, when the regression coefficient such as *β*_*G*_[*g*] is small $e^{\beta_{G}\left[g\right]}\approx 1+\beta_{G}\left[g\right]$, suggesting that 100 × *β*_*G*_[*g*] can be interpreted as the expected percentage change in the outcome due to gene editing. Similar interpretation applies to *β*_*T*_ and *β*_*TG*_[*g*].

In arrayed CRISPR screens, a large number of gene knockouts are applied to cells in different wells. However, not all these gene knockouts will affect the biomarker expression level, and the gene knockouts that do affect the biomarker expression level can have different effect sizes. To account for the between gene variation, we assume that *β*_*G*_[*g*] follows a mixture distribution:

$$\beta_{G}\left[g\right]∼\left(1-\theta \left[g\right]\right)\;\text{Normal}\left(0,\sigma_{\alpha }\right)+\theta \left[g\right]\;\text{Normal}\left(\mu_{\beta_{G}},\sigma_{\beta_{G}}\right)$$ (7)

*θ*[*g*] indicates whether gene *g* can truly affect the biomarker expression level (Yes: *θ*[*g*] = 1; No: *θ*[*g*] = 0). For genes that have no effect on the biomarker expression level, we assume that they follow a normal distribution Normal(0, *σ*_*α*_). *σ*_*α*_ represents the between gene variation among these negative genes. For genes that do have an effect on the biomarker expression level, we assume that they follow a normal distribution $\text{Normal}\left(\mu_{\beta_{G}},\sigma_{\beta_{G}}\right)$. $\mu_{\beta_{G}}$ represents the average effect of gene editing and can be interpreted as percentage change. $\sigma_{\beta_{G}}$ represents the between gene variation among these positive genes. Since interaction is also gene specific, we assume it follows a mixture distribution

$$\beta_{TG}\left[g\right]∼\theta \left[g\right]\;\text{Normal}\left(\mu_{\beta_{TG}},\sigma_{\beta_{TG}}\right)$$ (8)

$\mu_{\beta_{TG}}$ and $\sigma_{\beta_{TG}}$ to represent the average and between gene variability.

### Implementation

The above simulation model is implemented in an R package (https://github.com/DS-QuBi/arrayedCRISPRscreener). To simulate an arrayed CRISPR data set, one needs to provide inputs including a plate layout (which specifies what condition is applied to a particular well), the number of true hits, the desired number of cells in a single well ($\mu_{{\text{N}}_{\text{cell}}}$), spatial bias (Spatial Bias[*i*, *j*]), measurement error (*σ*_tech_), basal expression level (*α*), average gene editing effect ($\mu_{\beta_{G}}$), between gene variation for the genes that are true hits ($\sigma_{\beta_{G}}$) and true non-hits (*σ*_*α*_), as well as between cell variation (*σ*_*cell*_). The above simulation parameter values can come from literature, expert elicitation or estimation based on historical experimental data. The simulated arrayed CRISPR data are organized so that each row represents a well at *i*th row and *j*th column.

### The template data

To make sure our simulation model can generate similar arrayed CRISPR screening data as real ones, we used an in-house experimental arrayed CRISPR screen data set (unpublished work). There we measured the levels of *γ*-H2AX, a biomarker that indicates the presence of DNA damage. The screen was performed using the A549 lung adenocarcinoma cell model. Cells were seeded in a single 384-well plate. After 72 hours gRNA transfection, cells were fixed and stained. The images were then taken by using a confocal microscope. PerkinElmer’s Columbus was used for image analysis and quantified the fluorescent intensity of the *γ*-H2AX. Overall, the resulting data consisted of 359 wells from each 384-well plate, including 18 neutral controls, 69 genetic positive controls, and 272 different gene knockouts.

#### Code of ethics

The A549 Cas9 cell used in this project was derived from an original vial obtained from ATCC (CCL-185 Lot 62783414). The A549 Cas9 cell line tested negative for mycoplasma and the genotype was confirmed by STR Fingerprinting analysis.

### Analysis workflow

An arrayed CRISPR screen data analysis workflow includes a normalization and hit calling method. The biomarker expression values are first normalized to remove the spatial bias before being used for hit calling, where the gene knockouts that affect the biomarker expression levels are identified. In this study we investigated 6 example workflows consisted by 3 different normalization methods and 2 different hit calling methods.

#### Normalization methods

Normalization specifically means mitigation of spatial bias in the raw data and is not to be confused with data transformation. In this study, we investigated three different normalization scenarios: 1) No normalization, 2) LOESS normalization [8], and 3) B-score normalization [9]. Other normalization methods include Z-score normalization [25, 26], Control-Plate Regression (CPR) normalization [26], Well Correction [25], and GC-RMA normalization [27]. It is beyond the scope of this manuscript to evaluate all these normalization methods.

No normalization refers to the use of log-transformed data without any further adjustments for hit calling. LOESS normalization [8] is a standard method which addresses plate effects by fitting a local weighted regression model across all wells on a single plate. This approach effectively removes systematic variations while preserving biologically relevant variations. When working with a 384-well plate format, LOESS normalization can be applied to each plate individually to correct for within plate variation [28]. In contrast, B-score normalization [9] is another method used for normalization in the 384-well plate. It performs normalization plate by plate by using the median polish algorithm. It removes the row and column effects by subtracting the medians of each row and column from the measurement [28].

#### Hit calling algorithms

After normalization, the log transformed expression values are used for hit calling. The t test and linear mixed effect (LME) model [10] are two options for this purpose.

The t test compares the mean of tested gKO wells to the mean of the neutral control wells. We used the t.test() R function for the t test, comparing the log transformed biomarker expression values of gKO wells to those of neutral wells. We assumed unequal variances between the two groups and used Welch’s approximation for degrees of freedom. When applying t test to arrayed CRISPR data with multiple plates, we first performed between plate normalization by using the mean of neutral control wells in each plate.

When multiple plates are used in an arrayed CRISPR screening experiment, the linear mixed effect (LME) model [10] is useful to account for additional sources of variability such as between plate variability. In this study, we used the lmer function in the R package lme4 [10] with the model definition as ‘log(biomarker expression level) ~ gKO + (1|Plate)’. gKO corresponds to gene knockout and is treated as a fixed effect. In contrast, (1|Plate) corresponds to the random intercept due to different plates.

#### Performance evaluation

We evaluated each workflow with three metrics: true positive rate (TPR,$\text{TPR}=\frac{\text{TP}}{\text{TP}+\text{FN}}$), positive predictive value (PPV,$\text{PPV}=\frac{\text{TP}}{\text{TP}+\text{FP}}$) and F1-score ($\frac{2\text{PPV}\times \text{TPR}}{\text{PPV}+\text{TPR}}$). TP refers to the true positive. FN refers to the false negative. FP refers to the false positive. The TPR measures the proportion of actual positives that are correctly identified by the workflow, whereas PPV measures the proportion of predicted positives that are actually positive. F1-score is a measure of predictive performance, and it ranges from 0 to 1. When F1-score = 1, it indicates perfect precision and recall.

## Results

### Simulation model

**Fig 1:**
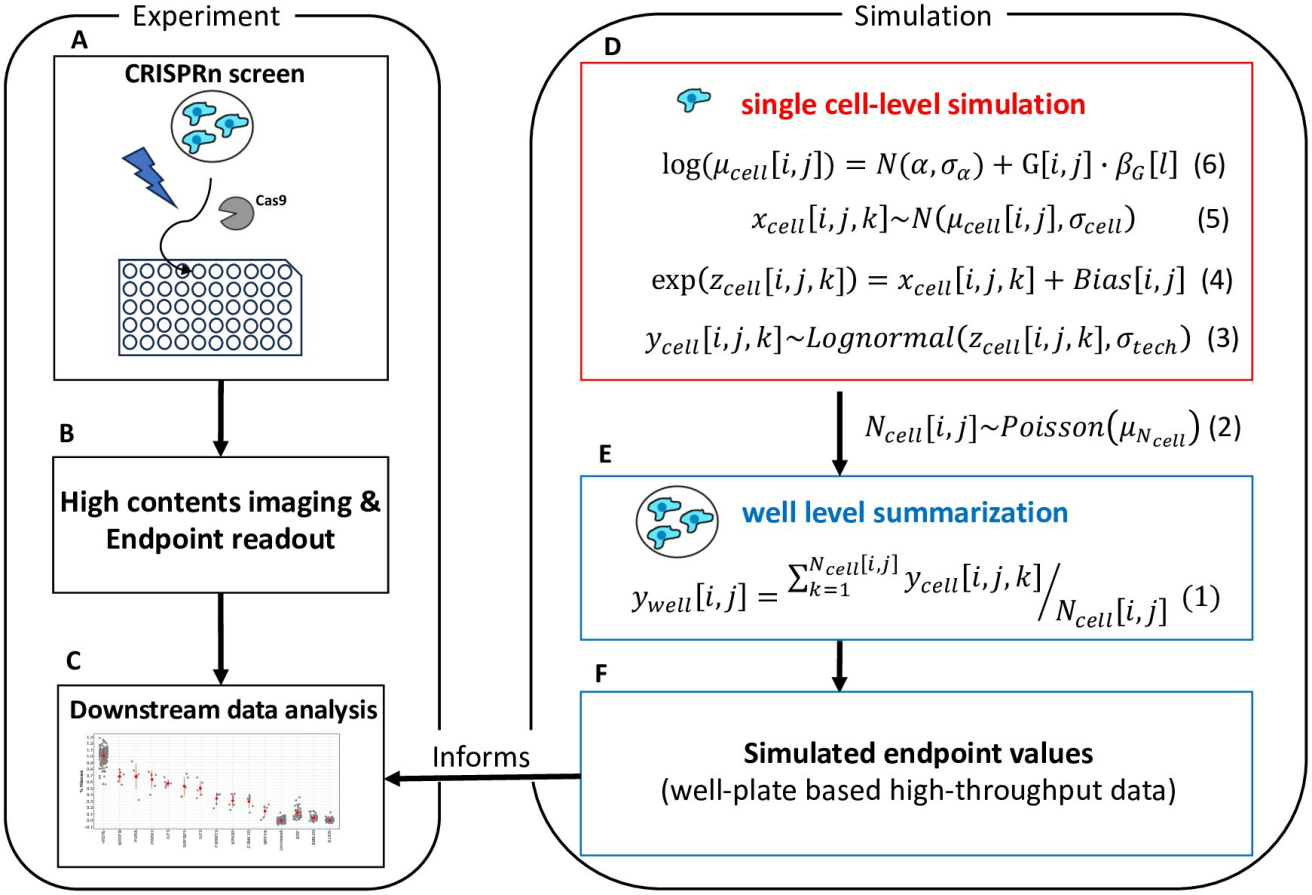
Overall simulation workflow. Experiment: (A) Plate based high-throughput arrayed CRISPR screen in which each genetic perturbation is applied in each well. (B) High-content imaging in which the endpoint readouts are a combination of multiple phenotypic and molecular biomarkers relevant for the mechanism of actions under study at the single-cell level. (C) Downstream data analysis for endpoint readouts including spatial normalization and hit callings. Simulation: the process consists of two main steps. (D) Single cell-level simulation incorporating genetic knockout effects, biological & technical variations, and spatial bias. (E) Well-level summarization of simulated endpoint values of cells in the well (e.g., mean, median, summation, etc.). (F) The simulated endpoint values is the same format of arrayed CRISPR screen pipeline which can be used as input to the downstream data analysis pipeline.

In this study, we developed a statistical simulation model that mimics the data-generating mechanism of arrayed CRISPR screen experiments, and allows us to simulate arrayed CRISPR screening data with known gene editing effect sizes, spatial bias, biological and technical variation (Fig 1). The mathematical details of the model were described in the method section and the full model is below:

$$y\left[i,j\right]=\frac{\sum_{k=1}^{{\text{N}}_{\text{cell}}\left[i,j\right]}y_{\text{per}\;\text{cell}}\left[i,j\right]\left[k\right]}{{\text{N}}_{\text{cell}}\left[i,j\right]}$$ (1)

$${\text{N}}_{\text{cell}}\left[i,j\right]∼\text{Poisson}\left(\mu_{{\text{N}}_{\text{cell}}}\right)$$ (2)

$$y_{\text{per}\;\text{cell}}\left[i,j\right]\left[k\right]∼\text{Lognormal}\left(z_{\text{per}\;\text{cell}}\left[i,j\right]\left[k\right],\sigma_{\text{tech}}\right)$$ (3)

$$exp\left(z_{\text{per}\;\text{cell}}\left[i,j\right]\left[k\right]\right)=x_{\text{per}\;\text{cell}}\left[i,j\right]\left[k\right]+\text{Spatial}\;\text{Bias}\left[i,j\right]$$ (4)

$$x_{\text{per}\;\text{cell}}\left[i,j\right]\left[k\right]∼\text{Normal}\left(\mu_{\text{per}\;\text{cell}}\left[i,j\right],\sigma_{\text{cell}}\right)$$ (5)

$$log\left(\mu_{\text{per}\;\text{cell}}\left[i,j\right]\right)=\alpha +\beta_{G}\left[g\right]\text{G}\left[i,j\right]+\left(\beta_{T}+\beta_{TG}\left[g\right]\text{G}\left[i,j\right]\right)\text{T}\left[i,j\right]$$ (6)

$$\beta_{G}\left[g\right]∼\left(1-\theta \left[g\right]\right)\;\text{Normal}\left(0,\sigma_{\alpha }\right)+\theta \left[g\right]\;\text{Normal}\left(\mu_{\beta_{G}},\sigma_{\beta_{G}}\right)$$ (7)

$$\beta_{TG}\left[g\right]∼\theta \left[g\right]\;\text{Normal}\left(\mu_{\beta_{TG}},\sigma_{\beta_{TG}}\right)$$ (8)

**Fig 2:**
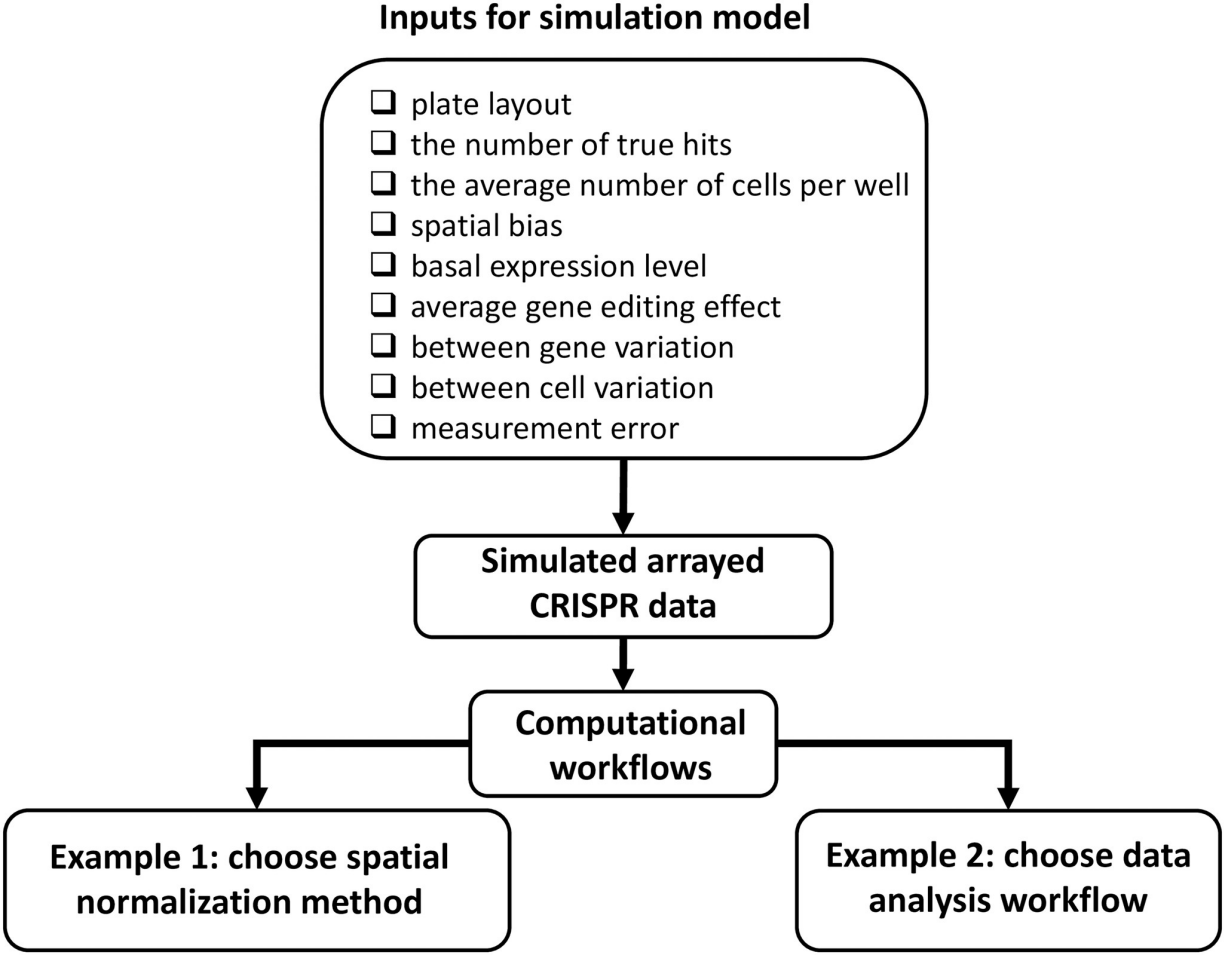
Benchmark workflow. The simulation model requires the following inputs: plate layout, the number of true hits, the average number of cells per well, spatial bias, basal expression level, average gene editing effect, between gene variation, between cell variation and measurement error. The simulated data set will be used as input for various computational workflows. A computation workflow includes a normalization step and a hit calling step. The example 1 shows how the simulation model helps to choose the spatial normalization method. The example 2 shows how simulation model helps to choose the data analysis workflow.

To simulate an arrayed CRISPR data set, one needs to specify a plate layout (which specifies what condition is applied to a particular well), the number of true hits, the average number of cells in a single well ($\mu_{{\text{N}}_{\text{cell}}}$), spatial bias, measurement error (*σ*_tech_), basal expression level (*α*), average gene editing effect (*β*_*G*_), between gene variation for the genes that are true hits ($\sigma_{\beta_{G}}$) and true non-hits (*σ*_*α*_), as well as between cell variation (*σ*_*cell*_) (Fig 2). The simulated data set is then processed to remove spatial bias before hit calling. In this work, we focus on 6 example workflows with three different normalization methods (no normalization, LOESS and B-score), and two different hit calling tools (t test and linear mixed effect model).

### Arrayed CRISPR screening data simulation

**Fig 3:**
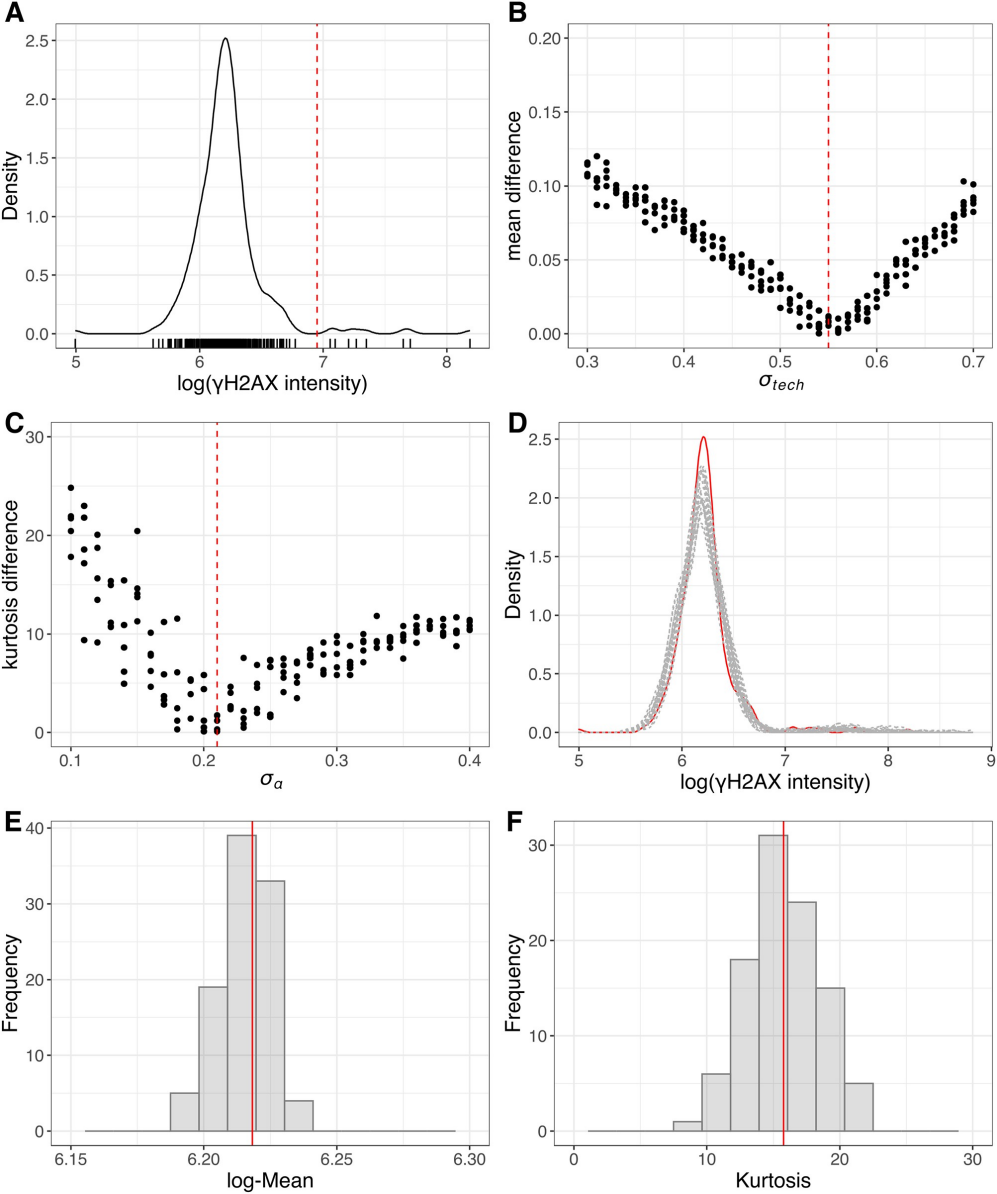
Comparison of simulated and real arrayed CRISPR screening data. (A) the distribution of real data. The red dashed line corresponds to the cutoff for hit calling. (B) x-axis represents the values of σ_tech and y-axis corresponds to the absolute difference of the mean values between real data and simulated data with given σ_tech. The red dash line represents the optimum value of σ_tech (C) x-axis represents the values of σ_α and y-axis corresponds to the absolute difference between the kurtosis values of real data and simulated data with given σ_α. The red dash line represents the optimum value of σ_α. (D) the distributions of simulated (grey) and real data (red) sets. For simulation, the estimated input parameters from figures (A-C) were used to generate 20 simulated data sets. (E) histogram of the mean values from 100 simulated data sets, with red line indicating the mean value of the real data. (F) histogram of the kurtosis values from 100 simulated data sets, with the red line indicating the kurtosis value of the real data.

To ensure our simulation model can generate realistic synthetic arrayed CRISPR screening data, we used an in-house arrayed CRISPR screening data (Fig 3A) to estimate input parameter values that dictate the simulation. These input parameters include *α* (the mean of the background values), *σ*_*α*_ (standard deviation of the background values), $\mu_{\beta_{\text{G}}}$(the mean effect size of the gKO treatments), $\sigma_{\beta_{\text{G}}}$(standard deviation of the effect size of the gKO treatments), *σ*_*tech*_ (standard deviation of measurement error), and $\mu_{{\text{N}}_{\text{cell}}}$ (the expected number of cells per well). Here, a “hit” was defined as a gene where the endpoint readout was greater than 2.5-fold increase over neutral control. We derived the values of *α* = 6.03 by calculating the average value from the wells that the log(*γ*-H2AX) values were below 6.95 (Fig 3A). Similarly, we derived the values of $\mu_{\beta_{\text{G}}}=1.41$ and $\sigma_{\beta_{\text{G}}}=0.38$ by calculating the mean and standard deviation from the wells that the log(*γ*-H2AX) values were above 6.95 (Fig 3A). The threshold log(*γ*-H2AX) value 6,95 corresponds to 2.5 folds higher expression compared to the neutral control, and 2.5 fold change is a stringent threshold to separate hits from non-hits so that distributional parameters can be estimated for each population separately. Furthermore, we derived the values of *σ*_*tech*_ = 0.55 and *σ*_*α*_ = 0.21 by minimizing the distance between the simulated data and real data in terms of the mean and kurtosis (a measure of the “tailedness” of the probability distribution) (Fig 3B&3C). $\mu_{{\text{N}}_{\text{cell}}}$ is set as 1131 which is the average number of cells per well in the example data set. The simulated data showed similar distribution as the real data (Fig 3D). To account for randomness of simulated data sets, we generated 100 simulated data sets. We observed that the simulated data sets consistently produced similar mean and kurtosis values compared to the real data (Fig 3E&3F).

### Example 1: Use the simulation model to choose spatial normalization method

**Fig 4:**
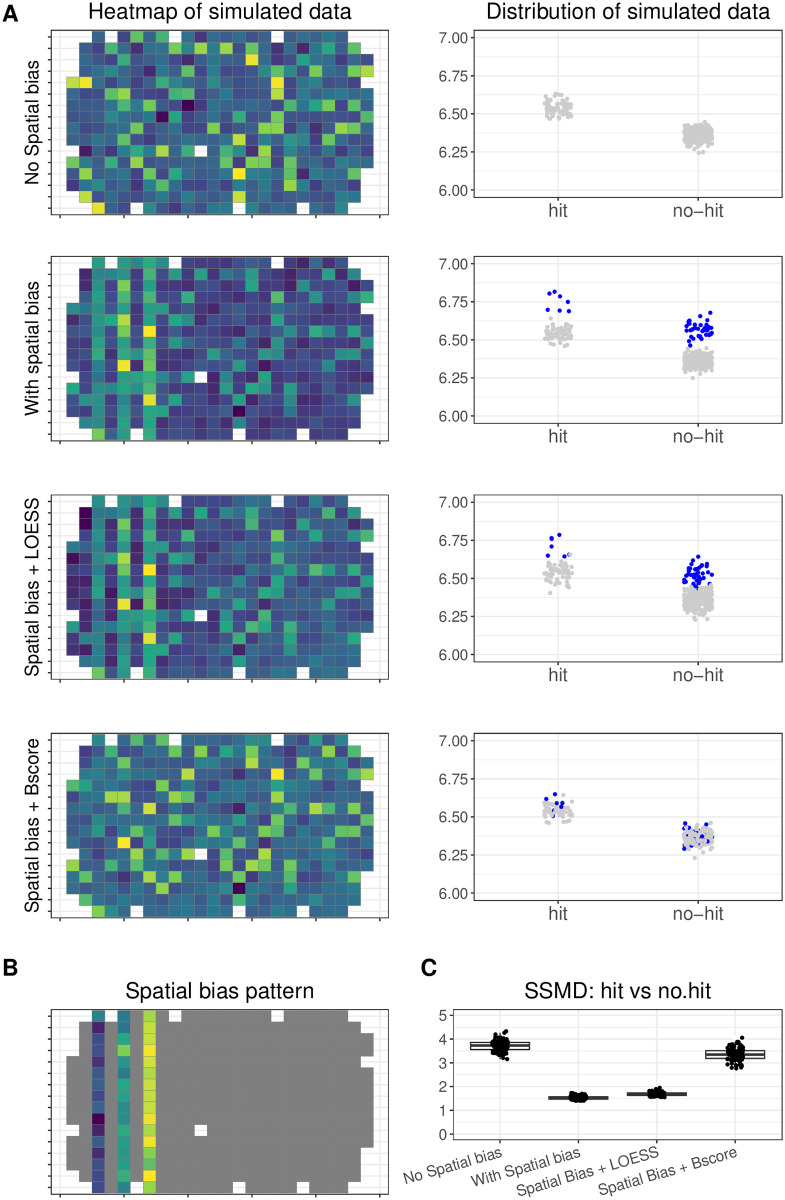
Assessment of spatial normalization methods. (A) shows the visualization of simulated datasets using heatmaps (1st column) and jitter plots (2nd column). The first row represents the simulated dataset without spatial bias. The second row represents the simulated datasets with spatial bias. Rows three and four represent the same bias-affected data set, after correction with LOESS and B-Score, respectively. (B) shows the pre-defined spatial bias added for the simulation where 3 columns were manually selected. (C) shows the distribution of SSMD values between simulated values of hits and no-hits. Each dot corresponds to a single simulation, and the simulation was repeated 100 time.

Spatial bias is one of the common technical artefacts affecting arrayed CRISPR screening data. With the simulation model, we generated an arrayed CRISPR data set for a single plate with known spatial bias (Fig 4). Wells from the columns 3, 5 and 7 were chosen to be affected by spatial bias (Fig 4B). In each simulation, we assumed that the spatial bias of individual wells from columns 3, 5 and 7 are randomly drawn from normal distribution *Normal*(83, 5), *Normal*(100, 5), and *Normal*(117, 5), respectively. 83, 100, 117 correspond to 20%, 24% and 28% of the average intensity value observed in the neutral control wells in the example data set, respectively. To mitigate the spatial effect, we applied either LOESS or B-score normalization to the simulated data. The strictly standardized mean difference (SSMD) was used to quantify the gene editing effect. Based on 100 simulated data sets, we observed that SSMD was smaller when there was spatial bias, and no normalization was applied. After B-score normalization, the SSMD increased to a level where it is comparable with there being no spatial bias. In contrast, SSMD did not change after LOESS normalization (Fig 4C).

### Example 2: Use the simulation model to choose data analysis workflow

**Fig 5:**
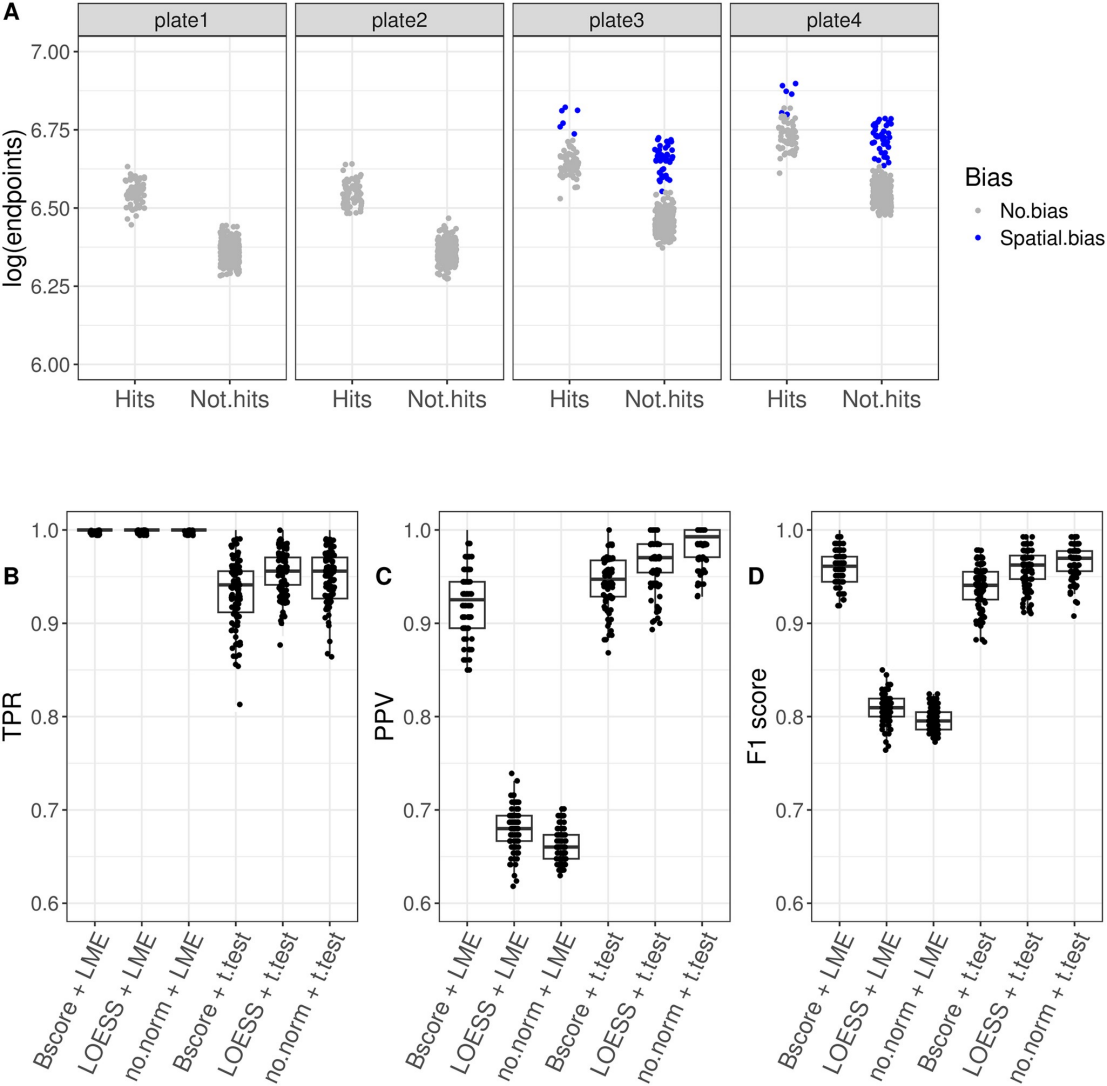
Assessment of hit calling pipelines. The figure (A) shows a single batch of simulated arrayed CRISPR screening data with 4 plates. Plate 3 and 4 were chosen to be affected by plate effects and spatial bias. The plate effect was generated by increasing the overall signals with pre-defined amount. The spatial bias was generated with the same approach as in Fig 4. Each dot represents the endpoint value of each well, and blue dots correspond to the one with spatial bias. The figures (B-D) show the distribution of True Positive Rates (TPR), Positive Predictive Value (PPV) and F1 score for 6 workflows. Each dot corresponds to TPR, PPV or F1 value for a simulated screen dataset of 4 plates, and the simulation was repeated 100 times.

In this study, we evaluated 6 example workflows including “No normalization + LME”, “LOESS + LME”, “B-score + LME”, “No normalization + t test”, “LOESS + t test”, “B-score + t test”. To assess their performance, we generated synthetic arrayed CRISPR screening data with four plates. Specifically, plate 3 and plate 4 were altered by spatial bias similar to the previous section (Fig 5A). In addition, plate 3 and plate 4 were also affected by batch effect, showing higher expression levels than plate 1 and plate 2 (Fig 5A). The batch effect was introduced by increasing the *α* (the mean of the background values) value. Specifically, for plate 1 and plate 2 we set *α* = 6.03. For plate 3 we set *α* = 6.12 corresponding to a 10% increase in background. For plate 4 we set *α* = 6.21 corresponding to a 20% increase. Each simulated data set was analyzed by 6 different workflows to identify gene editing hits. Our analysis revealed that the performance of these 6 workflow in terms of True Positive Rate (TPR) (Fig 5B), Positive Predictive Value (PPV) and F1 score (Fig 5C, Table 1).

**Table 1:** Performance of 6 example workflows.

Hit calling	Spatial normalisation	TPR	PPV	F1 score
LME	B score	1 ± 0	0.9241 ± 0.0673	0.960 ± 0.0365
	LOESS	1 ± 0	0.6791 ± 0.0428	0.809 ± 0.0304
	no.norm	1 ± 0	0.6611 ± 0.0342	0.796 ± 0.0247
t test	B score	0.934 ± 0.0721	0.9451 ± 0.0538	0.939 ± 0.0442
	LOESS	0.953 ± 0.0490	0.9661 ± 0.0547	0.959 ± 0.0402
	no.norm	0.949 ± 0.0525	0.9851 ± 0.0373	0.967 ± 0.0347

## Discussion

Arrayed CRISPR screen is a powerful tool for large-scale investigation of gene knockout effects [5, 29]. However, the analysis and interpretation of arrayed CRISPR screening data are often hindered by technical artefacts such as spatial biases within a plate and batch effects between plates [24]. As a result, normalization is used to mitigate these technical artefacts before progressing to hit calling. Many normalization and hit calling methods are available, but the choice of them is often made based on the subjective preference of data analysts rather than a data-driven fashion. To help scientists to make principled choices, we developed a statistical simulation model that can generate synthetic arrayed CRISPR screening data closely resembling the real data. Since the ground truth is known in the simulations, they are often used to evaluate computational workflows for high dimensional data, such as RNA sequencing [30, 31] and microbiome [32]. To the best of our knowledge, simulation approaches have not been used to benchmark computational workflows for arrayed CRISPR screen data but only for pooled CRISPR screen data [12]. Though simulations exist for pooled CRISPR screens, they cannot be used for arrayed CRISPR screens due to differences between the assays. For example, arrayed CRISPR screens commonly rely on high content imaging analysis, whereas pooled CRISPR screens typically use sequencing counts. Moreover, in an arrayed CRISPR screen, only one knockout is applied per well, whereas in a pooled CRISPR screen each well contains cells with different. Therefore, the eventual readout in the pooled CRISPR screen depends on not only the genes but also interactions between the cells with different knockouts. Simulation models for other high-throughput omics like RNAseq do not consider spatial bias. Therefore, it is necessary to have an arrayed CRISPR screen specific simulation model in order to evaluate normalization methods for removing spatial bias [12]. Our simulation model is the first effort that focuses on high content imaging readouts and can simulate arrayed CRISPR screen data with specified spatial bias, while accounting for variations between different genes, cells, wells and plates. This makes our simulation model useful for evaluating different computational workflows for diverse arrayed CRISPR screening experiments.

Our model specification (and its associated parameterization) is based on the arrayed CRISPR screen workflow used in AstraZeneca, and the noise properties that we believe each step to have. Specifically, each single cell has a fluorescent intensity that is captured in the image analysis. These intensity values correlate with the abundance of the protein biomarker. In the model we assumed that the variation of intensity values between cells can come from two sources, measurement error when measuring the intensity and cell-to-cell variation in the protein abundance. The measurement error (*σ*_tech_) refers to photon noise and read noise in fluorescence microscopy, and this parameter can be estimated when we have repeated measurements from the same cell. Since there are many cells in each well, we can also estimate the cell-to-cell variation (*σ*_cell_). To be able to identify both parameters simultaneously, we need to work with the single cell data with repeated measurements. We acknowledge that this is not always possible. When that is the case, these two parameters cannot be identified at the same time. For an instance, we cannot identify *σ*_tech_ and *σ*_cell_ independently based on the example data presented in this work. As a result, we fixed *σ*_cell_ = 0. In this case, *σ*_tech_ contains both cell-to-cell and technical variability. In addition to modeling different variations, we also modeled the treatment effect on the log scale since we can conveniently simulate effect size (e.g. 30% higher than neutral control) by setting *β*_*G*_ = 0.3. In contrast, we modelled the spatial bias on the original scale because we assumed that the spatial bias is additive by following a modeling tradition. We acknowledge that there is more than one way to parameterize the model and the assumptions we made may not hold for all situations. It is beyond the scope of this manuscript to investigate which model specification is the correct one. Therefore, users need to be cautious when they make decisions based on the simulations.

To use this model simulating arrayed CRISPR data, we first need to specify the values of model parameters. This can be done either by expert elicitation or by estimation based on historical data. In this study, we showed an example how one can estimate the parameter values based on historical data. In case that no historical data exist, scientists can use the default parameter values with the assumption that they can represent their own CRISPR screen. However, we acknowledge that this assumption may not hold, thus the users need to be cautious with the decision they make in that situation. We then used two examples to demonstrate that our simulation model can help make principled choices of normalization method to mitigate the spatial bias, and of data analysis workflow to maximize both sensitivity and accuracy. For the first example, a column-wise spatial bias scenario, we found that both B-score normalization or LOESS mitigated this bias. However, LOESS normalized out some of the gene-editing effect, and therefore B-score normalization is preferred. In the second example, we compared 6 example data analysis workflows across many simulated data sets and found that a combination of B-score normalization and LME achieved the best performance.

### Strength and limitations

Our simulation model is flexible, as it can be used to produce arrayed CRISPR screening data corresponding to any plate layout. Moreover, our simulation model considers cellular heterogeneity which can be overlooked in arrayed CRISPR screening data when only using well-level data (averaged over all the cells in a given well). A recent study [33] suggested that it was important to understand the role of cellular heterogeneity in deciphering the intricacies of gene knockout effects. In this study, we focused on an additive spatial bias that is characterized by column effects since this is the most common pattern that we have observed in practice. Recently, multiplicative spatial bias was suggested [22] and other forms of spatial bias have been reported [7]. To evaluate the performance of the normalization methods in those alternative scenarios, our simulation model can be expanded in the future studies to evaluate other spatial bias patterns and other biological treatment scenarios. In conclusion, our simulation model offers a powerful tool for making principled choices of analysis methods for arrayed CRISPR screening experiments.

## Supporting information

S1 File Template data and all R codes for reproducing the results. (ZIP)
