# Correction: A statistical simulation model to guide the choices of analytical methods in arrayed CRISPR screen experiments

**DOI:** 10.1371/journal.pone.0320884

**Published:** 2025-03-20

**Authors:** Chang Sik Kim, Jonathan Cairns, Valentina Quarantotti, Bogumil Kaczkowski, Yinhai Wang, Peter Konings, Xiang Zhang

There are errors in the Author Contributions. The correct contributions are:

Conceptualization: Chang Sik Kim, Yinhai Wang, Peter Konings, Xiang Zhang

Formal analysis: Chang Sik Kim

Investigation: Chang Sik Kim, Valentina Quarantotti, Xiang Zhang

Methodology: Xiang Zhang

Project administration: Xiang Zhang

Resources: Bogumil Kaczkowski, Yinhai Wang, Peter Konings

Software: Chang Sik Kim

Supervision: Bogumil Kaczkowski, Yinhai Wang, Peter Konings

Visualization: Chang Sik Kim

Writing – original draft: Chang Sik Kim, Valentina Quarantotti, Xiang Zhang

Writing – review & editing: Chang Sik Kim, Jonathan Cairns, Valentina Quarantotti, Bogumil Kaczkowski, Yinhai Wang, Peter Konings, Xiang Zhang

In the Implementation subsection of the Methods, there is an error in the first sentence of the first paragraph. The correct sentence is: The above simulation model is implemented in an R package (https://github.com/AstraZeneca/arrayedCRISPRscreener).

The affiliation for the sixth and seventh author is incorrect. The correct affiliation is not indicated. Peter Konings and Xiang Zhang are not affiliated with #1. Peter Konings and Xiang Zhang are affiliated with Data Sciences & Quantitative Biology, Discovery Sciences, BioPharmaceuticals R&D, AstraZeneca, Gothenburg, Sweden.
