# Supplementary figures and images for: A statistical simulation model to guide the choices of analytical methods in arrayed CRISPR screen experiments
Source: PLoS One. 2024 Aug 20;19(8):e0307445. doi: 10.1371/journal.pone.0307445 (PMC11335118; doi:10.1371/journal.pone.0307445)

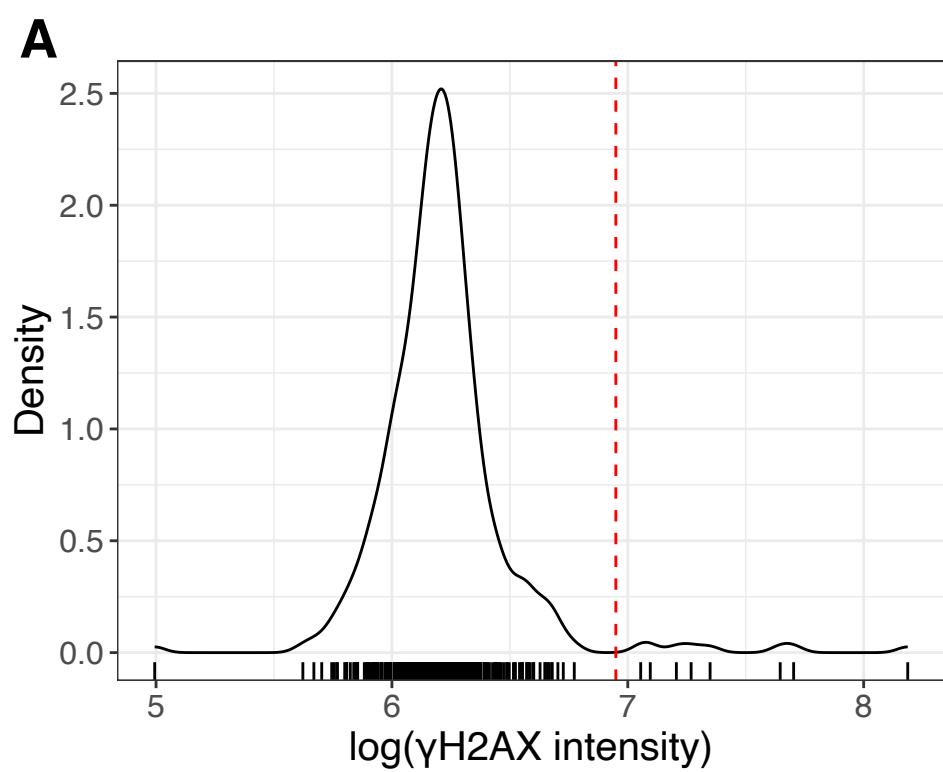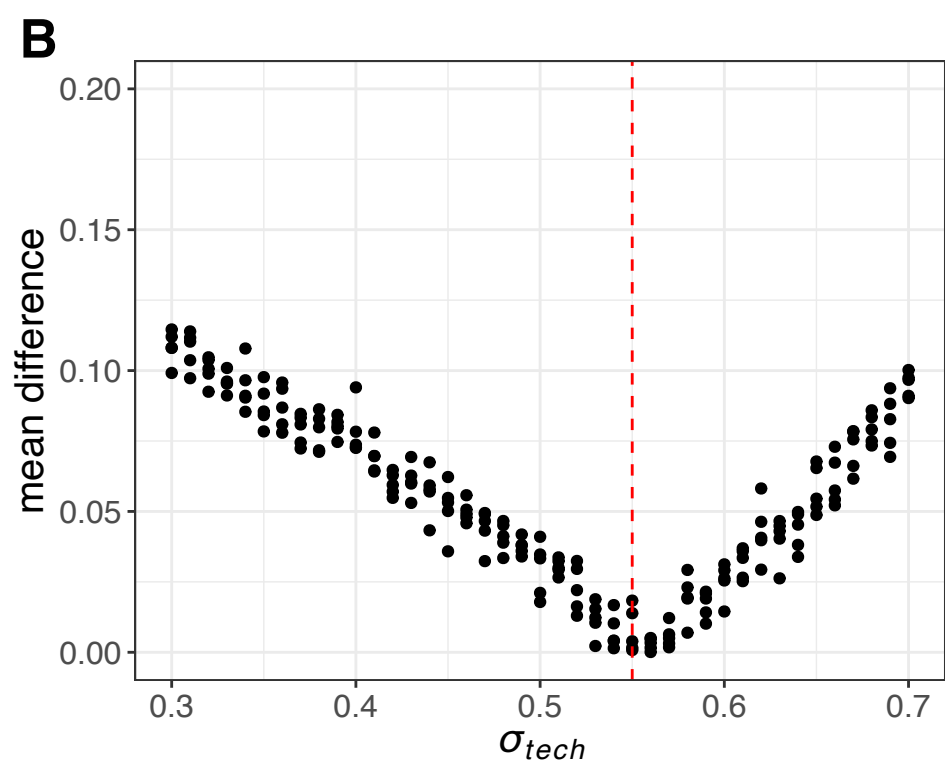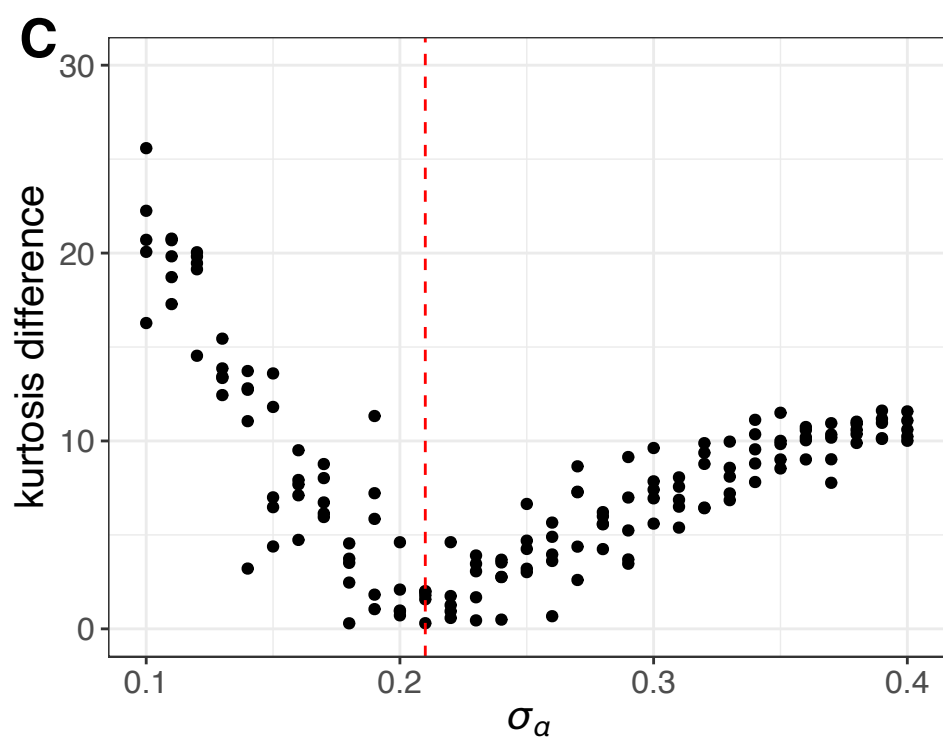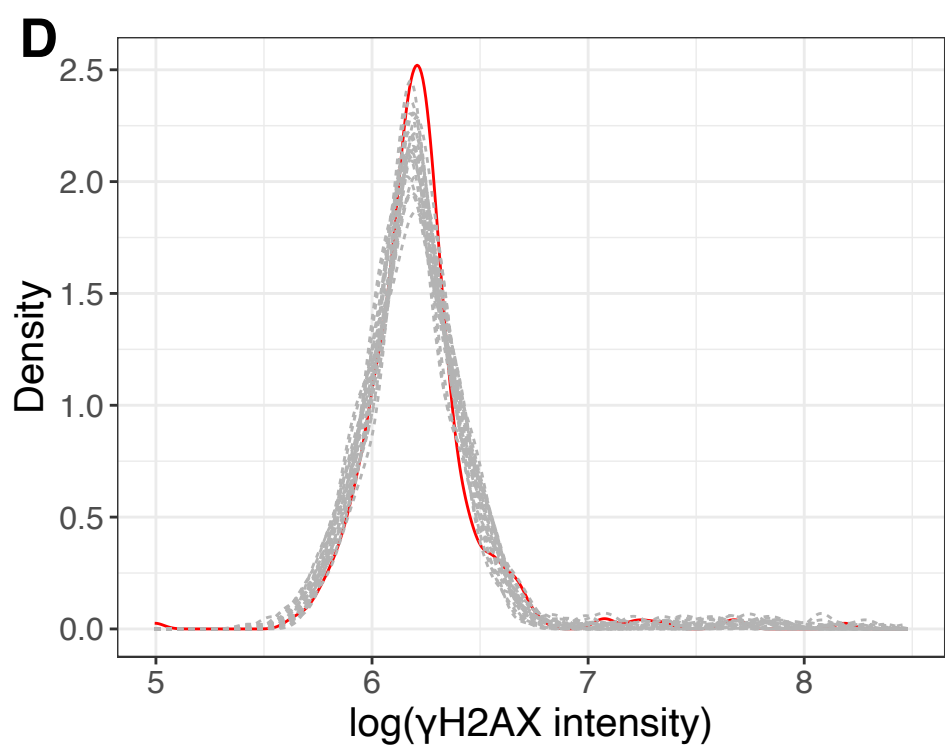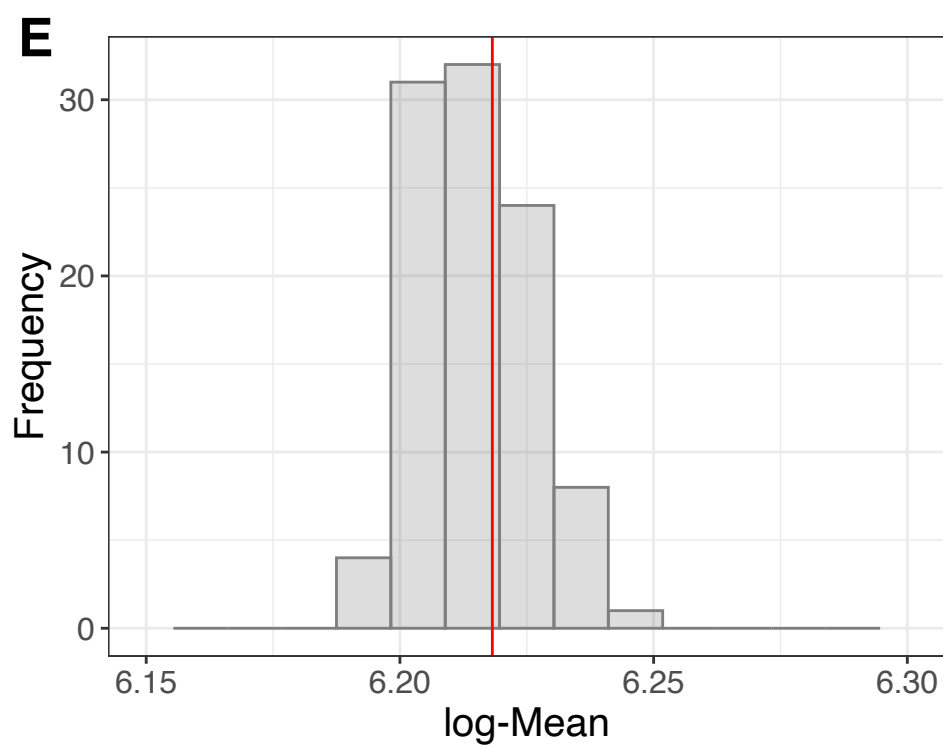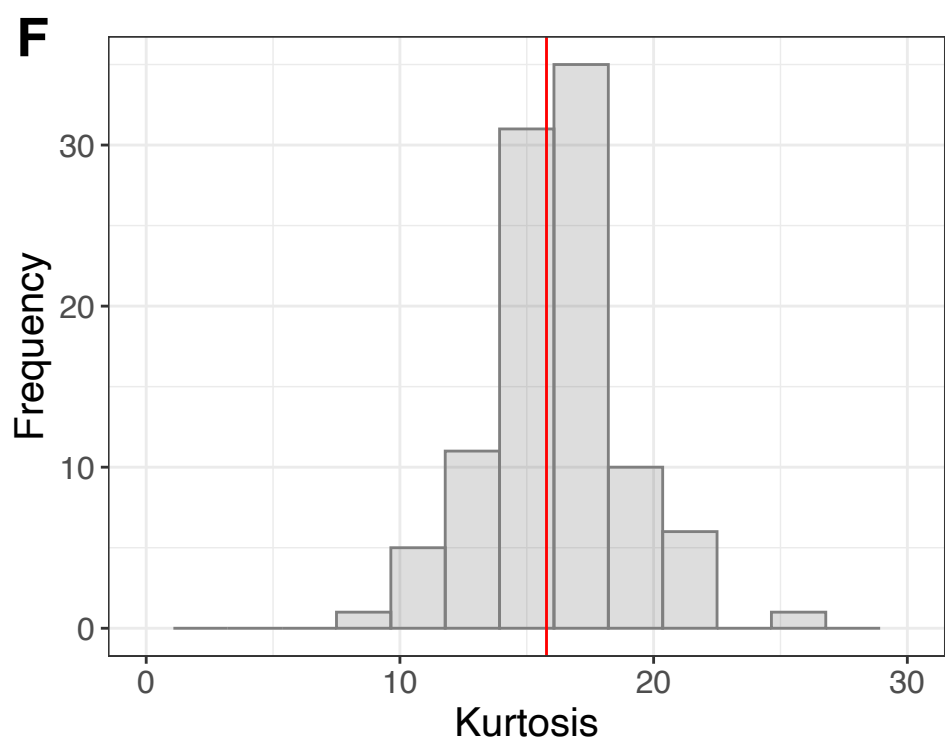

Supplement: S1 File — (ZIP) [file pone.0307445.s001.zip › Scripts_Manuscript/Figure3_manuscript.pdf]

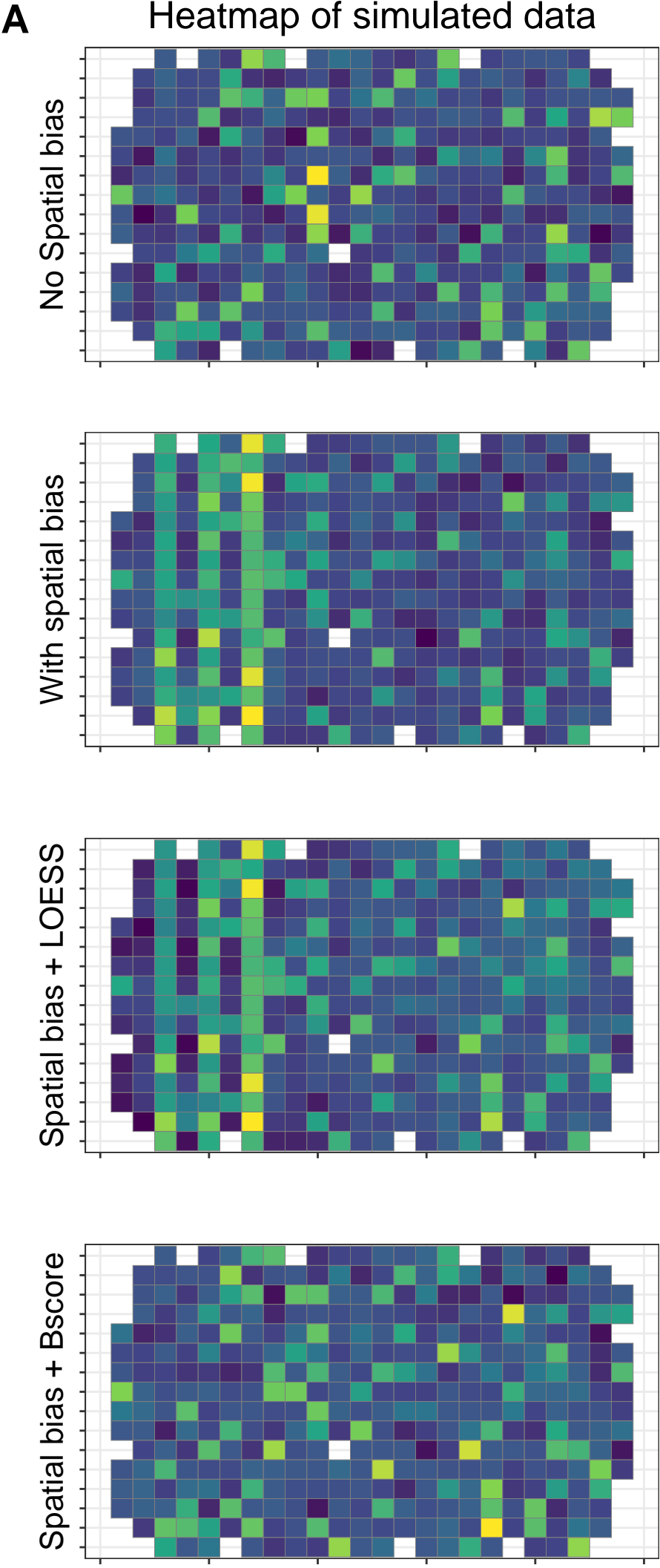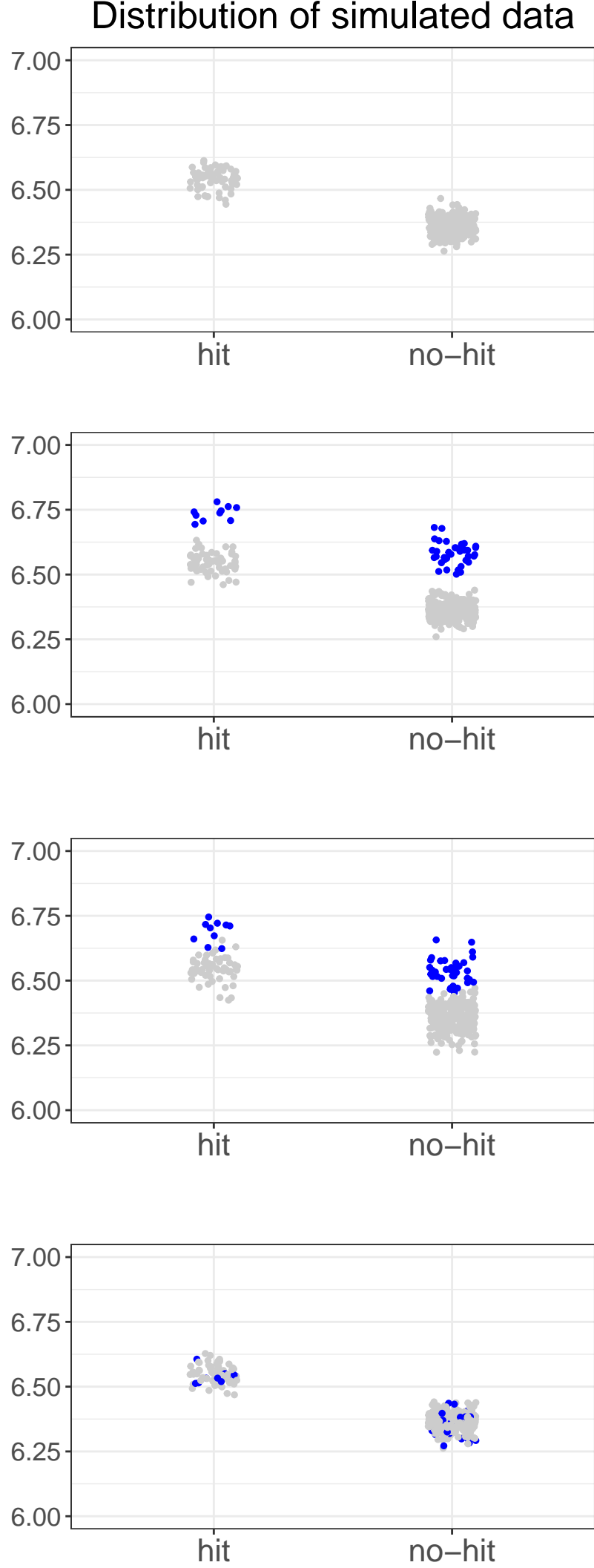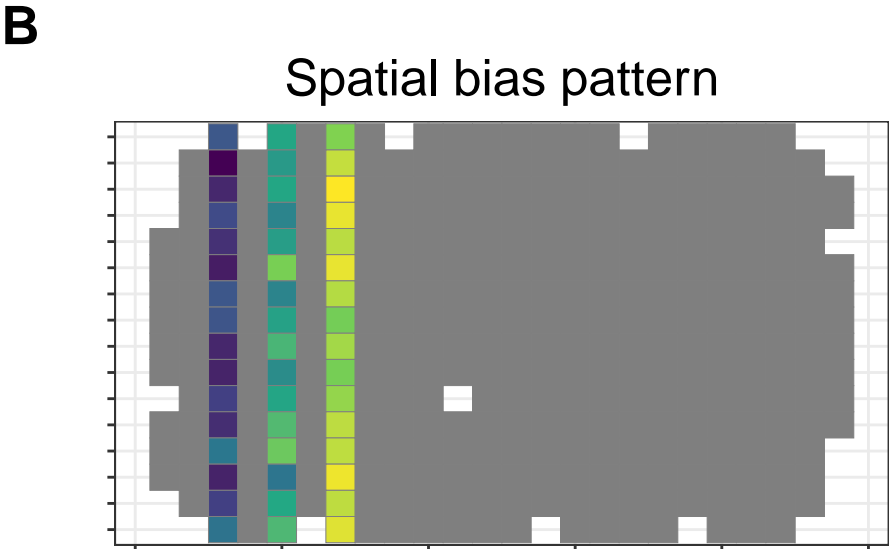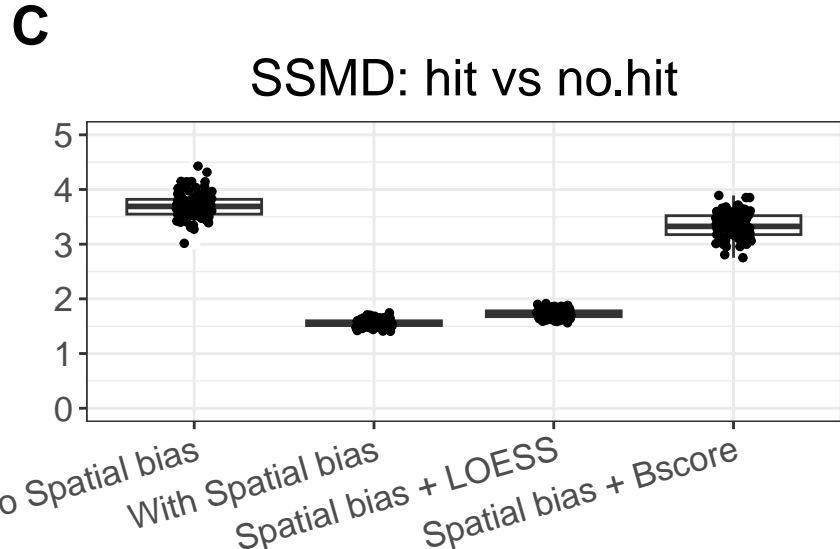

Supplement: S1 File — (ZIP) [file pone.0307445.s001.zip › Scripts_Manuscript/Figure4_manuscript.pdf]

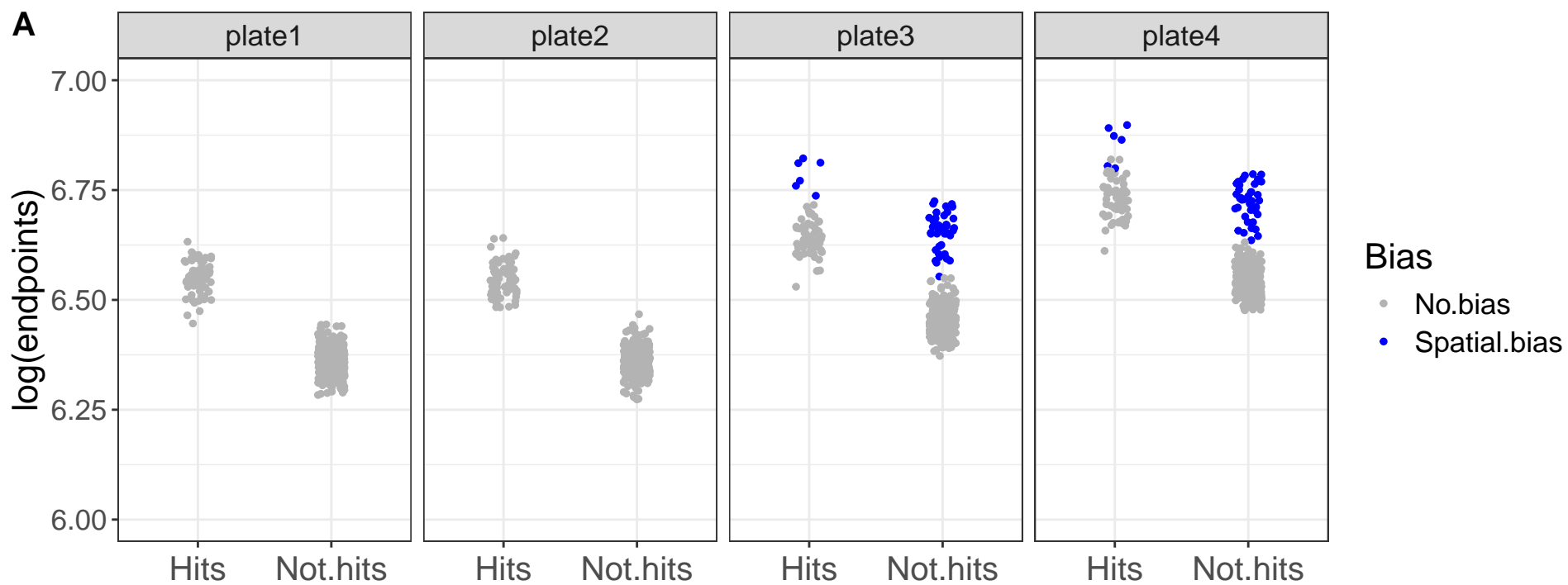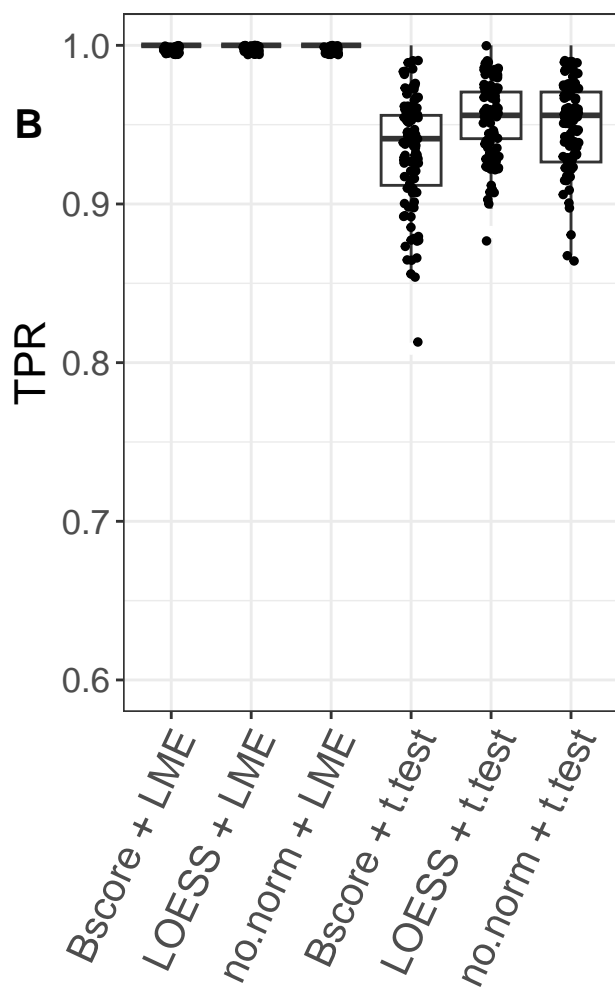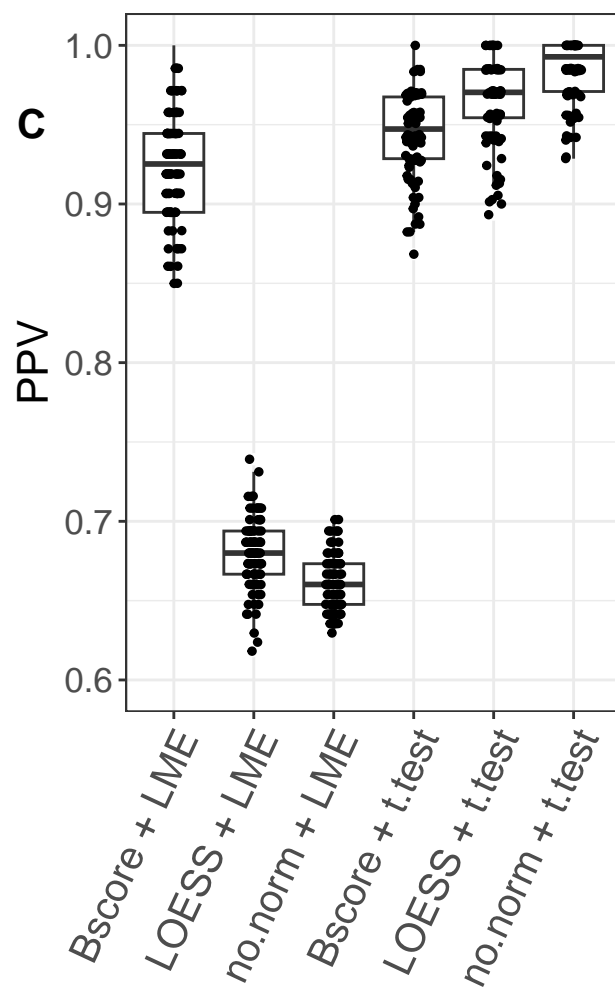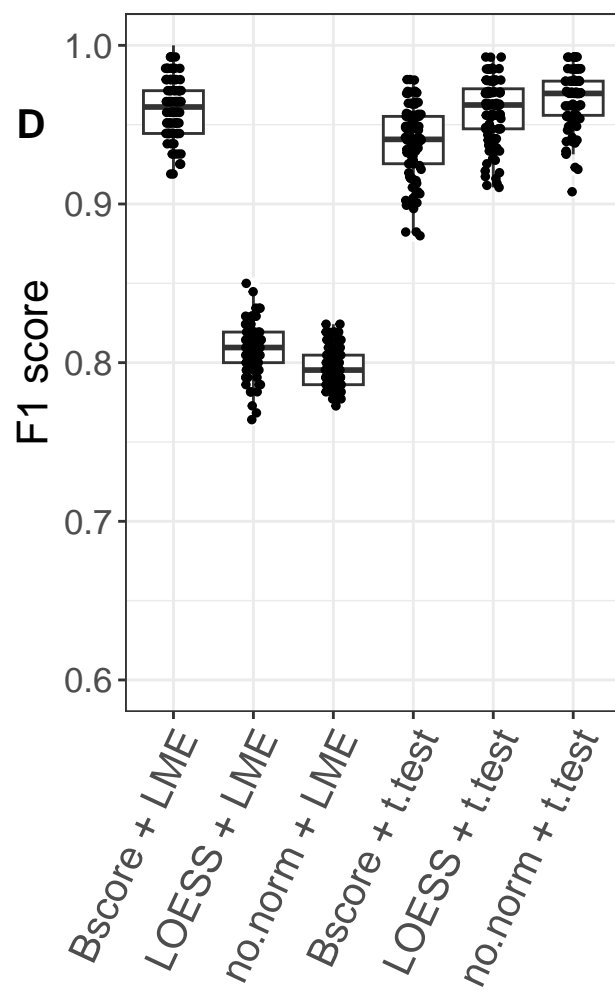

Supplement: S1 File — (ZIP) [file pone.0307445.s001.zip › Scripts_Manuscript/Figure5_manuscript.pdf]
